# Frequency of Low Birth Weight and its Relationship With Maternal Nutritional and Dietary Factors: A Cross-Sectional Study

**DOI:** 10.7759/cureus.8731

**Published:** 2020-06-21

**Authors:** Saira Jamshed, Farah - Khan, Amna Begum, Beena Barkat Ali, Zuneera Akram, Madiha Ariff

**Affiliations:** 1 Obstetrics and Gynecology, Hamdard University Hospital, Karachi, PAK; 2 Obstetrics and Gynecology, Karachi Medical and Dental College/Abbasi Shaheed Hospital, Karachi, PAK; 3 Obstetrics and Gynecology, Agha Khan Hospital for Women, Karachi, PAK; 4 Pharmacology, Baqai Institute of Pharmaceutical Sciences, Baqai Medical University, Karachi, PAK; 5 Internal Medicine, Dow University of Health Sciences, Karachi, PAK

**Keywords:** low birth weight, mothers, diets, association, cross-sectional study

## Abstract

Background: Low birth weight (LBW) is linked with fetal and neonatal mortality and morbidity; also it can slow down growth and cognitive development. Several factors contribute to pregnancy outcomes, including LBW deliveries, maternal nutrition, and additional dietary intake. Our study was aimed to identify the frequency and factors associated with LBW mothers in Karachi, Pakistan.

Methods: A cross-sectional survey was carried out at the Obstetrics and Gynaecological Department of Hamdard Hospital, Karachi. A total of 195 healthy pregnant females were enrolled. All the relevant data were collected from March 1st, 2019 to August 31st, 2019 with the help of a structured questionnaire designed specifically for the study where mothers were also asked about consumption of iron, vitamin, and extra meals since they became pregnant. Written, informed consent was taken from all of the participants before data collection. Data analysis was performed using a statistical package for the social sciences (SPSS) version 20.0. A Chi-square test was used for checking associations between the studied maternal factors and the weight of the children.

Results: There was a total of 195 pregnant females selected for the study. The frequency of LBW infants was found to be 57 (29.2%) in these women. The mean age of the females was 29.29 ± 5.22 years, 142 (72.8%) of them had a body mass index (BMI) of 25.0 kg/m^2^ or more, 102 (52.3%) of them had hemoglobin (Hb) between 10 and 11 mg/dL. The study results further showed that maternal Hb (p=0.02), vitamin C intake (p=0.037), iron intake (p=0.01), and consumption of extra meals during pregnancy (p=0.021) were significantly associated with a LBW of the children. Mothers whose Hb <10 mg/dL, no intake of vitamin C, or iron, and extra meals during pregnancy were more likely to have a child with LBW than others.

Conclusion: It can be concluded that maternal nutritional and dietary factors are very important during fetal development, and they have a significant relationship with the birth weight.

## Introduction

According to the World Health Organization (WHO), low birth weight (LBW) is defined as the first weight after birth which is less than 2500 g (5.5 pounds), resulting from preterm birth (birth before 37 completed weeks) or due to intrauterine growth restriction or from both [1-2]. Birth weight not only predicts the health status of the mother but also gives future information about the survival, development, and long-term health of the baby. LBW is one of the important primary determinant and contributor to neonatal and infant mortality that contributes for nearly half of all perinatal and one-third of all infant deaths [3-4]. LBW babies have 40 times more chances to die within the first 30 days of life as compared to those with normal birth weight (NBW); so considering its importance, LBW has been used as an important health indicator of a public health problem as per directives of WHO, because it reflects the malnutrition and metabolic conditions of the mother, as well as poor health care at population level during pregnancy, hence, it is the strongest predictor of newborn health and survival [1, 5-7]. Developing countries of the world have been facing a very high burden of this condition with a 16.5% frequency of LBW, in comparison to developed countries (7%). Specifically, a condition in South Asia is worse due to low socioeconomic status (SES), and poor health care during pregnancy; as the global estimate is 18 million babies born per year with LBW and 9.3 million (50% of total) of them belong to South Asia [2]. However, Pakistan has made some progress in achieving Millennium Development Goals with a frequency of 12%-25%, specifically in Lahore 21%, and in Karachi 29% babies born with LBW by the year 2017 and 2018, respectively [8-10]. 

As we know, the nutritional needs during pregnancy are higher than usual days of life, and proper balance with extra nutrients can help both mother and fetus in protecting long-term health outcomes. Many physiological modifications in mother, as well as the growth of fetus, increase the metabolic demand, and inadequate maternal nutritional status showed adverse pregnancy outcomes, poor infant survival, and impaired intellectual development in the coming life [11-12]. Previous studies showed that the women who had consumed junk food, imbalanced diet, consumed less quantity of food, or those who escape meals during the first eight-week period of pregnancy are more prone to maternal mortality as well as fetal morbidity and mortality as compared to those who took healthily and enough quantity of meals. Hence, it is postulated that a well-fed mother gives birth to a healthy and normal weight child because they had a more favorable intrauterine environment [13]. There are many risk factors, for LBW including poor maternal nutrition and lifestyle factors such as the use of alcohol and tobacco; pregnancy complications such as hypertension, low socio-economic conditions, maternal age, maternal body composition, and parity [1,14]. Maternal health and nutritional status are modifiable risk factors that are particularly crucial in determining infant birth weight [1]. The intake of sufficient energy, protein, and nutrients to meet maternal and fetal requirements is required for optimal growth and birth weight, low nutritional diet, and inadequate weight gain during pregnancy contribute to fewer nutrients that are required for fetal growth, such as vitamins and iron. Iron promotes the formation of new hemoglobin (Hb) and is responsible for an adequate supply of energy and oxygen to body organs [15-16]. Maternal anemia can develop when Hb levels drop below 11 g/dL, which may lead to the unavailability of iron in an extracellular environment for erythropoiesis [17]. This study was conducted to identify the frequency of LBW in the women of Karachi, Pakistan, and to explore the relationship of LBW with maternal nutritional and dietary factors among them. 

## Materials and methods

A cross-sectional survey was carried out at the Obstetrics and Gynecological Department of Hamdard University Hospital, Karachi, among 195 healthy pregnant females. A nonprobability convenient sampling technique was used in this study. The ethical approval was taken from the institutional review board of the hospital. Taking the percentage frequency of the study outcome as 50% for the most liberal estimate, with a 95% confidence level and 7.5% precision, the minimum calculated sample size was 171 pregnant females. Healthy pregnant females with either singleton or multiple pregnancies with at least three antenatal checkups were included while females giving birth at gestational age less than 34 weeks or above 41 weeks and 6 days, or with a complicated pregnancy were excluded from the study. Written, informed consent was taken from all of the participants before data collection. No incentive, monetary or otherwise, was offered to the study participants. All the relevant data were collected from March 1st, 2019 to August 31st, 2019 with the help of a structured questionnaire designed specifically for the study. Data were analyzed using the Statistical Package for Social Sciences (SPSS) version 20.0. For descriptive analysis, means and standard deviations were calculated for continuous variables while frequencies and percentages were generated for categorical variables. Inferential analysis was performed using the chi-square test to check associations between the maternal factors and the weight of the children whereas the significance level was kept at 0.05 (p-value).

## Results

There was a total of 195 pregnant females selected for the study. The frequency of LBW infants was found to be 57(29.2%) in these women. The mean age of the females was 29.29 ± 5.22 years, 142(72.8%) of them had a body mass index (BMI) of 25.0 kg/m2 or more, 102(52.3%) of them had Hb between 10 and 11 mg/dL, 172(88.2%) of them used to take each of vitamin C and iron during pregnancy, 136(69.7%) of them consumed extra meals during pregnancy while only 5(2.6%) of them were tobacco smokers/chewers (Table 1). 

**Table 1:** Participants profile. BMI, body mass index; SD, standard deviation

Variables (n=195)	Mean±SD/Frequency (%)
Birth weight (kg)	
Up to 2.5	57 (29.2)
More than 2.5	138 (70.8)
Maternal age (years)	29.29±5.22
Maternal BMI (kg/m^2^) before pregnancy	
Up to 24.9	53 (27.2)
25.0 or more	142 (72.8)
Maternal hemoglobin (mg/dL)	
<10	44 (22.6)
10 to 11	102 (52.3)
>11	49 (25.1)
Vitamin C intake	
Yes	172 (88.2)
No	23 (11.8)
Iron intake	
Yes	172 (88.2)
No	23 (11.8)
Extra meals consumed	
Yes	136 (69.7)
No	59 (30.3)
Tobacco smoking/chewing	
Yes	5 (2.6)
No	190 (97.4)

The study results further showed that most of the neonates 20(45.5%) with weight less than 2.5 kg were having the maternal Hb of less than 10 mg/dL, whereas 39(79.6%) of neonates were related to the mothers with Hb of >11 mg/dL, with a significant association (p=0.02). It was also revealed that vitamin C intake (p=0.037), iron intake (p=0.01), and consumption of extra meals during pregnancy (p=0.021) were significantly associated with a LBW of the children, where mothers whose Hb <10 mg/dL, no intake of vitamin C, or iron, and extra meals during pregnancy were more likely to have a child with LBW than others (Table 2). 

**Table 2:** Relationship of maternal nutritional and dietary factors with birth weight. BMI, body mass index *p-value <0.05 is considered significant

Variables	Birth weight (kg)		p-value*
	Up to 2.5 kg (n=57)	More than 2.5kg (n=138)	
	n (%)	n (%)	
BMI (kg/m^2^) before pregnancy			
Up to 24.9	20 (37.7)	33 (63.2)	0.111
25.0 or more	37 (26.1)	105 (73.9)	
Hemoglobin (mg/dL)			
<10	20 (45.5)	24 (54.5)	0.02*
10 to 11	27 (26.5)	75 (73.5)	
>11	10 (20.4)	39 (79.6)	
Vitamin C intake			
Yes	46 (26.7)	126 (73.3)	0.037*
No	11 (47.8)	12 (52.2)	
Iron intake			
Yes	45 (26.2)	127 (73.8)	0.01*
No	12 (52.2)	11 (47.8)	
Extra meals consumed			
Yes	33 (24.3)	103 (75.7)	0.021*
No	24 (40.7)	35 (59.3)	
Tobacco smoking/chewing			
Yes	2 (40.0)	3 (60.0)	0.631
No	55 (28.9)	135 (71.1)	

## Discussion

The current study was carried out to find out the frequency of LBW deliveries and to understand the effect of certain maternal factors on the birth weight of infants. According to regional statistics, the global burden of neonatal mortality was found to be high in low- and middle-income countries (LMICs), where almost all deaths account for LBW cases [5]. There are several associated risk factors for LBW like poor maternal nutrition, anemia, smoking, primiparity, low SES, mental stress during pregnancy, abuse in the family, lack of antenatal visits, short maternal height, and low maternal weight [18]. A study from Botswana revealed that some other factors may also be linked with LBW such as maternal illiteracy, being unmarried, and late or less or no availability of ANC services, and place of birth, while in Egypt, low maternal education was the only factor associated with LBW [19-20]. However, a study from Nigeria depicted that maternal predictors of LBW were marital status, occupation, residential accommodation, and lack of ANC services, hypertension and related diseases, antepartum hemorrhage, and intrauterine growth restriction (IUGR) [21]. In this study, the frequency of LBW was found to be 29.2%. This study finding is comparable with another study also conducted in Karachi, Pakistan, and found that 29% of women gave birth to LBW babies [10]. We found that mothers whose BMI was less than 25 kg/m2 were more likely to deliver LBW babies. In the present study, women with normal BMI gave birth to 73.9% NBW babies and 26.1% LBW babies. This finding was consistent with previous studies where the frequency of LBW babies was significantly higher among mothers with BMI <18.5 kg/m2 [22-24]. Another similar study described that lower BMI (<18 kg/m2) doubles the probability of LBW deliveries [25]. The lower value of BMI indicates that mothers were under-nourished which ultimately restricts the growth and development of a fetus in the uterus. It was observed in the present study that females with normal Hb levels are more likely to deliver NBW, and that there were 79.6% mothers with normal Hb level gave birth to NBW and 20.4% LBW babies. Similarly, those women with a very low Hb level, almost 45.5% were LBW deliverers, hence, it is confirmed that maternal low Hb is significantly associated with LBW. Our results are consistent with a previous study where low maternal Hb was significantly associated with LBW [26]. Imbalance or low dietary intake during pregnancy had adverse effects on birth outcomes [27-28]. It has been observed that in LMICs, women during pregnancy are at high risk of dietary and nutrient deficiencies, including iron, folic acid, iodine, zinc, vitamins A and D, riboflavin, B6 and B12; that is why developing countries especially in Asia, have a high prevalence of LBW deliveries [12, 28-29]. Our findings were also not far from these results; we observed that the women who were taking iron and vitamin C supplements, as well as extra meals during pregnancy, had delivered a lower number of LBW babies and vice versa. It was indicated that the serum concentration of maternal vitamin C was significantly related to birth weight and length in case of full-term deliveries and that the 1 µg/mL increase in the serum vitamin C level led to a 27.2 g increase in birth weight and 0.17 cm increase in birth length. It was also shown that levels of vitamins C and E when compared together with birth weight and length, heaviest birth weight and the longest birth length were associated with the highest levels of vitamin C/upper vitamin E [30]. In light of our study, maternal nutritional and dietary factors were found to be very important for fetal development, and they play a significant role in the birth weight of a newborn. Although the study is not spared from bias, this study may have selection bias due to sampling technique used in it,

## Conclusions

The frequency of LBW infants was 29.2% in this study, which is very alarming because this is the future cause of neonatal death. In this study, we observe that BMI, Hb levels, vitamin C, iron intake, and extra meal intake were the factors associated with LBW deliveries. Hence, the maternal factors mentioned above should be considered important while striving to achieve a good birth weight of infants before and during pregnancy. If we make efforts to prevent or reduce LBW by takings steps and improving maternal nutritional and dietary intake, we will be able to reduce neonatal death. Policy makers may also consider the need for time for quality healthcare, nutrition education, and lifestyle changes in LBW high-risk mothers. 
